# Association of oral health knowledge, attitudes, and practice with dental caries status among 6–12-year-old Iranian orphaned children using the CAST index: A Cross-sectional study

**DOI:** 10.1371/journal.pone.0332983

**Published:** 2026-07-02

**Authors:** Zahra Jahanbakhti, Zahra Momeni

**Affiliations:** 1 Zahra Jahanbakhti – Student Research Committee, Alborz University of Medical Sciences, Karaj, Iran; 2 Zahra Momeni – Community Oral Health Department, School of Dentistry, Alborz University of Medical Sciences, Karaj, Iran; Shahid Beheshti University of Medical Sciences School of Dentistry, IRAN, ISLAMIC REPUBLIC OF

## Abstract

**Introduction:**

Orphaned children face heightened vulnerability due to the absence of parental care and limited access to preventive services. Evidence linking oral health knowledge, attitudes, and practice (KAP) with clinical outcomes in this population remains limited. This study aimed to assess oral health KAP among orphaned children in Karaj, Iran, and examine their associations with clinical indicators of dental caries, gingival status, and oral hygiene.

**Methods:**

In this cross-sectional study, 72 children aged 6–12 years residing in four government-run quasi-family centers in Karaj were examined between October 2024 and January 2025 through a census sampling approach. Inclusion required age eligibility and informed consent, whereas children with systemic or developmental disorders or those receiving orthodontic treatment were excluded. Data were collected using a structured, validated questionnaire (α = 0.83) to assess KAP and demographic factors. Clinical examinations were performed by a calibrated examiner (ZJ) (Kappa = 87.68%) using the CAST, GI, and OHI-s indices. Logistic and linear regression models were used to examine predictor variables of KAP and oral health outcomes.

**Results:**

The mean scores of knowledge, attitude, and practice were 3.16 ± 1.60 (out of 7), 33.52 ± 4.36 (out of 50), and 10.26 ± 2.72 (out of 20), respectively. Overall, 62.5% of children demonstrated fair oral hygiene (OHI-s = 1.75 ± 1.58) with mild gingival inflammation. CAST assessment indicated that fewer than one-third of primary molars were sound, while more than half of permanent first molars showed enamel caries. Regression analyses showed that frequent toothbrushing (p = 0.015, OR=0.52, 95% CI: 0.30–0.88) and more positive attitudes toward oral health (p = 0.013, OR=0.70, 95% CI: 0.53–0.93) were significant predictors of improved oral status, whereas knowledge and self-reported practice were not consistent predictors.

**Conclusions:**

Orphaned children in Karaj demonstrated moderate oral hygiene, a high prevalence of untreated dental caries, and limited awareness of oral health. Addressing these behavioral and systemic gaps through targeted, evidence-based interventions—particularly oral health education, caregiver involvement, and routine dental monitoring—may help improve oral health outcomes in this vulnerable population.

## Introduction

Extensive scientific research has consistently highlighted the pivotal role of oral health in maintaining systemic well-being. Oral health is now recognized not merely as an element of dental care, but as a foundational determinant of overall health and quality of life [1]. Despite its critical importance, oral health remains one of the most frequently overlooked aspects of general health. Often referred to as a “silent epidemic,” poor oral health affects approximately 4 billion individuals worldwide, resulting in substantial economic and social burdens [2].

Global disparities in oral health disproportionately affect socially and economically disadvantaged groups. Among vulnerable populations, orphaned children represent a particularly high-risk group. Asia reports the highest number of orphaned children globally [3]. An orphan is defined as a child who has lost one or both parents [4]. Parental presence is critical not only for emotional and physical development but also for promoting and modeling health-related behaviors. The absence of family care has profound implications for both their general and oral health outcomes [5].

From the perspective of Iranian mothers, food preferences, high sugar consumption, reluctance to seek a doctor, and lack of self-efficacy in brushing teeth are the main child-related barriers to maintaining and improving children’s oral health [6]. Children living in orphanages often do not receive adequate education, guidance, or supervision regarding oral hygiene, which parents typically provide during the early developmental years [3]. As a result, their accumulated unmet oral health needs further widen health disparities among this population [7].

Numerous studies have demonstrated that dental caries in children are influenced by a wide range of factors, including sociodemographic status, behavioral patterns, and material deprivation [8]. A cross-sectional study among Iraqi schoolchildren [9] reported similar associations, reinforcing the importance of examining these determinants across different populations and contexts. Khattab et al. (2023) reported significantly higher prevalence and severity of dental caries among orphaned children compared to their school-going peers, emphasizing the urgent need for targeted, sustainable, and evidence-based preventive oral health strategies for this vulnerable population [5]. Similarly, a study by Kumari et al. (2021) found a high prevalence of gingival and periodontal diseases among orphaned children, findings largely attributed to inadequate oral hygiene practices and the absence of parental guidance or caregiver involvement [10]. Additionally, a recent systematic review by Gamal-AbdelNaser et al. (2024) reported a high risk of dental caries among orphaned children, underscoring their increased vulnerability. The authors further emphasized the need for continuous monitoring and periodic assessment of this population to support the development of effective interventions to improve their oral health status [11].

The high prevalence of oral disease in orphaned children [5], absence of parental support to educate in early preventive behaviors [12], economic barriers limit access to oral healthcare services for socially disadvantaged groups [7] and the lifelong benefits of establishing healthy habits during childhood [13] collectively emphasize the importance of prioritizing the assessment of the oral health status of orphaned children.

Conventional indices such as DMFT/dmft are widely used in caries surveillance; however, they do not adequately capture the full clinical burden of untreated disease, including pain, infection, and functional impact [13]. Although more recent systems, such as ICDAS [14] and PUFA/pufa [15], provide greater diagnostic sensitivity, important limitations remain. To address these gaps, Franken et al. (2011) introduced the CAST index [16], which classifies the complete spectrum of caries progression—from early non-cavitated lesions to advanced stages with clinical complications. This comprehensive framework aligns with contemporary caries management approaches, particularly in resource-limited settings where access to dental care is constrained. The CAST index has demonstrated strong applicability in global oral health research and has been increasingly implemented across diverse populations.

Although several studies have assessed the oral health status of orphaned children, few have explored the behavioral and attitudinal factors underlying these outcomes. Research using validated KAP instruments among orphans remains limited, especially in developing Asian countries. In response to this need, the present study aimed to evaluate the knowledge, attitudes, and practices related to oral health of orphaned children aged 6–12 years, providing a holistic understanding of the factors influencing their oral health outcomes.

## Materials and methods

### Study design and population

This study adhered to the STROBE guidelines for reporting observational research [14]. This cross-sectional study was conducted in Karaj, the capital of Alborz Province, located northwest of Tehran, which is Iran’s fourth most populous city, with around 1.6 million residents (2016 census) [15]. It is renowned as “Little Iran” for its immigrant-friendly and culturally diverse community [16]. Karaj hosts several government-run quasi-family centers accommodating children from diverse backgrounds, making it suitable for the study objectives.

All 72 children aged 6–12 years residing in government-run quasi-family residential centers (three boys’ centers and one girls’ center) in Karaj, Iran, were enrolled through census sampling. Data collection was conducted over four months, from October 2024 to January 2025.

The inclusion criteria were as follows: obtaining informed consent from participants or their legal guardians at the centers and being between 6 and 12 years of age. Children were excluded if they had systemic diseases, mental or physical disabilities, or were undergoing orthodontic treatment. Additionally, children who failed to cooperate during the clinical examination were excluded from the analysis. However, none of the eligible children met the exclusion criteria, and all 72 participants were included in the final analysis.

### Data collection

Data were collected through a structured questionnaire that included sections on demographic characteristics, dietary habits, and oral health practices. The questionnaire took approximately 20 minutes to complete. The children filled out the questionnaire as a self-report. However, for the younger children (ages 6–8), questions were read aloud in simplified language to ensure understanding. This adaptation was applied uniformly across participants to minimize misunderstanding while maintaining the original meaning of the questions. All questionnaires were completed and returned in the presence of the principal investigator (ZJ), providing no missing or incomplete data (response rate: 100%).

### Study instruments

This tool was adapted from the World Health Organization (WHO) standardized oral health questionnaire [17], which has previously been translated, culturally adapted, and validated in Iran [18]. The original WHO questionnaire, successfully pilot-tested in diverse countries worldwide, has previously been used with 6- and 12-year-old Iranian children [19]. The questionnaire included demographic information (age, gender, and school grade), history of receiving oral health education and dental visits, and assessment of oral hygiene practices. The children’s knowledge, attitude, and practices were assessed as outcome variables.

Furthermore, a second questionnaire was designed based on the validated framework proposed by Samiee Roudi et al. [20] to evaluate participants’ knowledge, attitudes, and practices concerning oral health. The face and content validity of this instrument have been previously established and discussed in earlier studies [20]. The questionnaire was developed by drawing upon authoritative references, including academic textbooks and peer‑reviewed scientific literature. It has been reviewed and refined with input from subject-matter experts and educational supervisors. The test-retest has been carried out on 25 research subjects (10 intervals) to test reliability. These subjects were excluded from the main study. Internal consistency of the variables has been assessed and confirmed using Cronbach’s alpha coefficient (α = 0.83).

Knowledge was assessed using seven yes/no questions covering basic oral health concepts, including the difference between primary and permanent teeth, the preventive roles of tooth brushing, dental floss, fluoride mouthwash, and a healthy diet. A correct (“Yes”) response was scored as 1, while “No” and “I don’t know” were scored as 0. The total knowledge score ranged from 0 to 7.

Attitudes were evaluated with 10 items rated on a five-point Likert scale (ranging from “Strongly Disagree” to “Strongly Agree”), producing scores between 10 and 50. This section examined participants’ attitudes toward oral hygiene and healthy dietary habits. Items 1 and 7 were reverse-scored to maintain consistency.

Practice was measured using four items on a five-point Likert scale (“Never” to “Always”), generating a total score ranging from 4 to 20. These items evaluated the frequency of toothbrushing, consumption of sugary snacks and beverages, and intake of fruits and vegetables.

Higher scores in all sections reflected more favorable knowledge, attitudes, and practices.

### Clinical examination

For calibration, the researcher (ZJ) performed clinical oral examination on 20 children under the supervision of an expert (intra-examiner kappa: 87.68). These children were not included in the study population. Then, the trained examiner (ZJ) assessed each participant using standard dental diagnostic tools, including a dental mirror, explorer, and WHO periodontal probe under natural lighting, with the child seated in a regular chair. Gingival Index (GI), Simplified Oral Hygiene Index (OHI-S) were recorded.

Also, the Caries Assessment Spectrum and Treatment (CAST) index records dental caries (ranging from 0 to 9) [21]. The coding system is defined as follows: code 0 indicates a sound tooth surface; code 1 refers to pits and/or fissures sealed with a sealant material; code 2 represents restored cavities with direct or indirect restorative material; code 3 denotes distinct visual changes in enamel; code 4 indicates discoloration suggestive of dentinal caries; code 5 refers to cavitated lesions involving dentine; code 6 reflects pulp involvement; code 7 indicates the presence of swelling or sinus tract associated with infected pulp; code 8 represents a tooth extracted due to caries-related complications; and code 9 includes other conditions not classified under the above categories. For analytical purposes, these codes were grouped according to the classification proposed by Baginska et al. [22] as follows: “healthy” (codes 0–2), “pre-morbidity” (code 3), “morbidity” (codes 4–5), “serious morbidity” (codes 6–7), “mortality” (code 8), and “None of the above” (code 9).

The oral hygiene indices were interpreted using the following thresholds [23]: DI-S and CI-S: 0.0–0.6: Good, 0.7–0.8: Fair, and 0.9–3.0: Poor; OHI-S: 0.0–1.2: Good oral hygiene, 1.3–3.0: Fair oral hygiene, and 3.1–6.0: Poor oral hygiene. And the Gingival index (GI) is as follows [24]: 0.1–1: Mild gingivitis, 0.1–2: Moderate gingivitis, 2.1–3: Severe gingivitis. All findings were documented using a standardized examination form.

### Statistical analyses

Data were analyzed using IBM SPSS Statistics for Windows, Version 27.0 (IBM Corp., Armonk, NY, USA). Descriptive statistics (mean, standard deviation, frequency, and percentage) were used to summarize demographic variables, oral health indices, and questionnaire scores.

Binary logistic regression was performed with dental caries status (categorized based on CAST scores: presence vs. absence of caries) as the dependent variable. A CAST score of ≥3 was considered the presence of caries. Linear regression analysis was used to assess associations between oral hygiene status (OHI-S scores) and independent variables. Covariates included age, gender, and oral health knowledge, attitude, and practice (KAP) scores. Both unadjusted and adjusted models were examined, with adjusted models controlling for age and gender. Adjusted odds ratios (OR) and 95% confidence intervals (CIs) were reported. A p-value < 0.05 was considered statistically significant.

### Research ethics

Before the start of the study, the objectives, procedures, and assessment tools were thoroughly explained to the centers’ supervisors and the children. Participation was voluntary, and written informed consent was obtained from all participants or their guardians at the centers. Also, supplementary details were provided in the questionnaire introduction. The completion served as a reaffirmation of the participants’ informed consent and voluntary participation in the study. The study protocol was reviewed and approved by the Ethics Committee of Alborz University of Medical Sciences (IR.ABZUMS.REC.1403.072). All procedures were conducted in accordance with the Declaration of Helsinki.

## Results

### Demographic characteristics

The study included 72 orphaned children aged 6–12 years residing in Karaj, Iran (mean age: 9.8 ± 1.87 years). Of these, 53 children (73.6%) reported at least one previous dental visit, and 28 children (38.9%) had received some form of oral health education.

Regarding self-perceived oral health status, 37 children (51.4%) rated their teeth as good, while 29 children (40.3%) reported good gingival health. Within the past 12 months, 13 children (18.1%) had experienced a toothache, and 29 (40.3%) had visited a dentist.

Notably, of those who reported a dental visit, 43 children (59.7%) were unaware of the specific reason for their most recent visit. In contrast, 13 children (18.1%) reported the visit was due to dental pain or issues related to their teeth, gums, or oral cavity. Additional demographic and background characteristics of the study population are summarized in Table 1.

**Table 1:** Demographic characteristics of 6- to 12-year-old orphaned children (n = 72).

Variable	N	(%)
**Gender**		
Girl	22	30.6
Boy	50	69.4
**History of receiving health education services**		
Yes	28	38.9
No	44	61.1
**History of visiting the dentist**		
Yes	53	73.6
No	19	26.4
**Tooth brushing**		
Never	0	0
Less than once a day	23	32
Once a day	31	43.1
Twice a day or more	18	25
**Use of fluoride toothpaste**		
Yes	67	93.1
No	5	6.9
**Snacks**		
About twice or more times a day	40	55.5
Less than twice a day	32	44.5

### Questionnaire

The mean (± standard deviation) scores for the children’s oral health knowledge, attitude, and practice were 3.16 ± 1.60 (total score ranged from 0 to 7), 33.52 ± 4.36 (total score ranged from 10 to 50), and 10.26 ± 2.72 (total score ranged from 4 to 20), respectively.

**Fig 1:**
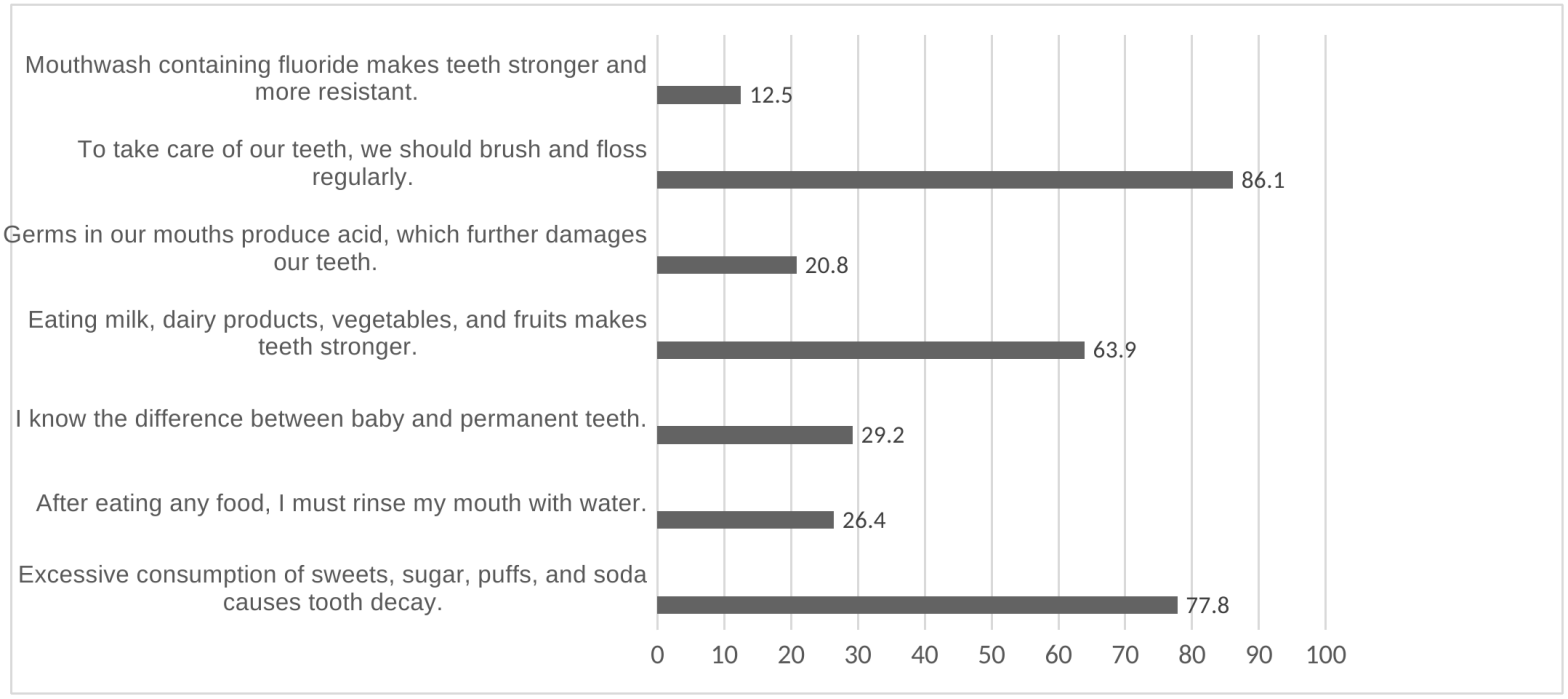
Percentage of correct responses to oral health knowledge questions among orphaned children aged 6 to 12 years (n = 72).

Fig 1 illustrates the percentage of correct responses to oral health knowledge questions. The majority of participants (86.1%) correctly identified that regular brushing and flossing are crucial for maintaining good dental health. More than 60% of children were aware that a healthy diet—including milk, dairy products, fruits, and vegetables—contributes to stronger teeth, and that excessive intake of sweets, sugary snacks, and soda causes tooth decay (77.8%). However, over 65% were unaware of the benefits of fluoride mouthwash, only 29.2% of children understood the difference between primary and permanent teeth, and just 20.8% knew that oral bacteria produce acids that damage teeth.

Table 2 summarizes participants’ attitudes toward oral health. 98.6% agreed that having healthy and attractive teeth is important, and 88.9% supported the habit of brushing after meals. Additionally, 66.7% strongly agreed that failure to brush leads to quicker tooth decay. However, 25% disagreed that flossing is the best way to clean between teeth, and nearly half (47.2%) believed that toothbrushes cannot clean all parts of the teeth.

**Table 2:** Distribution of orphaned children’s responses to oral health attitudes questions (n = 72).

Question	Strongly disagree		Disagree		Neutral		Agree		Strongly agree	
	N	(%)	N	(%)	N	(%)	N	(%)	N	(%)
I have a great interest in eating sweets, chocolate, and snacks.	0	0	15	20.8	12	6.7	25	34.7	20	27.8
Daily milk consumption is beneficial for dental health.	4	5.6	17	23.6	6	8.3	32	44.4	13	18.1
If we don’t brush our teeth, tooth decay will occur sooner.	0	0	3	4.2	5	6.9	48	66.7	16	22.2
A toothbrush alone does not clean all parts of the teeth.	18	25	34	47.2	8	11.1	11	15.3	1	1.4
Dental floss is the best way to clean the spaces between your teeth.	0	0	14	19.4	18	25	32	44.4	8	11.1
Bad breath is caused by not brushing and flossing regularly.	1	1.4	13	18.1	11	15.3	43	59.7	4	5.6
Using mouthwash containing fluoride can cause teeth to turn yellow and ugly.	2	2.8	12	6.7	43	59.7	14	19.4	1	1.4
Having beautiful, healthy teeth is important to me.	0	0	0	0	1	1.4	33	45.8	38	52.8
I need to get into the habit of brushing my teeth after eating.	1	1.4	12	16.7	3	4.2	43	59.7	13	18.1
I have faith in flossing at least once a day.	0	0	21	29.2	19	26.4	26	36.1	6	8.3

Table 3 presents the frequency of oral health-related practices. Only 12.5% of children reported consistently brushing and flossing properly, 43.1% did so occasionally, and 20.8% rarely engaged. Nearly half (47.2%) have never rinsed their mouths after consuming sweets. Furthermore, while 58.3% seldom consumed dairy products daily, over 40% reported occasionally or often eating fruits and vegetables.

**Table 3:** Distribution of orphaned children’s responses to oral health practice questions (n = 72).

Question	Never		Rarely		Sometimes		Often		Always	
	N	(%)	N	(%)	N	(%)	N	(%)	N	(%)
I brush and floss my teeth regularly and properly.	0	0	15	20.8	31	43.1	17	23.6	9	12.5
I always rinse my mouth after eating sweets and snacks.	34	47.2	19	26.4	11	15.3	6	8.3	2	2.8
I consume milk and dairy products every day.	3	4.2	42	58.3	23	31.9	2	2.8	2	2.8
I eat vegetables and fruits daily.	3	4.2	31	43.1	30	41.7	5	6.9	3	4.2

### Clinical examination

Table 4 presents the distribution of the oral health indices. The mean Gingival Index (GI) was 0.49 ± 0.51, indicating mild gingival inflammation. The mean Debris Index-Simplified (DI-S) was 1.48 ± 0.51, and the Calculus Index-Simplified (CI-S) was 0.26 ± 0.51. The overall Oral Hygiene Index-Simplified (OHI-S) score showed a broader distribution, with 62.5% in the fair category, 30.6% in good, and 6.9% in poor, yielding a mean score of 1.75 ± 0.76. These findings reflect moderate levels of plaque and debris among the study population.

**Table 4:** Qualitative distribution of oral hygiene indices (OHI-s) and mean and standard deviation of GI among orphaned children (n = 72).

Variable	Mean	Standard deviation	Good		Fair		Poor	
			(%)	N	(%)	N	(%)	N
DI-s	1.48	0.51	2.8	2	6.9	5	90.3	65
CI-s	0.26	0.51	86.1	62	4.2	3	9.7	7
OHI-s	1.75	0.76	30.6	22	62.5	45	6.9	5
**Variable**	**Mean**	**Standard deviation**	**Mild**		**Moderate**		**Severe**	
			**(%)**	N	**(%)**	N	**(%)**	N
GI	0.49	0.51	86.1	62	13.9	10	0	0

### Dental caries status based on the CAST index

#### Primary teeth.

**Fig 2:**
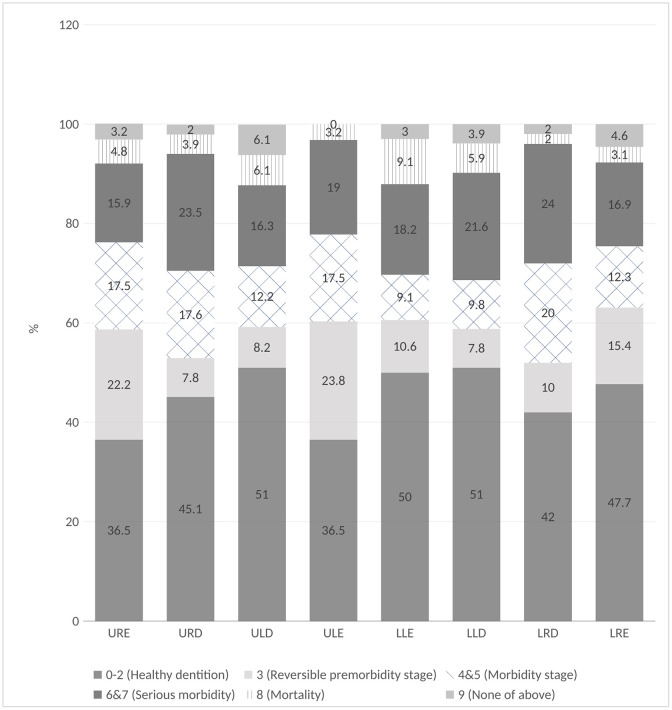
Distribution of the primary molars’ status based on CAST categories: healthy, pre-morbidity, morbidity, serious morbidity, and mortality.

In primary dentition, the highest frequency of sound teeth (code 0) was recorded for the upper right and left D teeth (teeth 54 and 64), both at 29.2%. The lowest prevalence of healthy primary molars was seen in the lower right D tooth (tooth 84), with only 18.1% classified as code 0. Across all primary teeth, no cases of sealant application (code 1) were documented. The prevalence of cavitated dentine lesions (code 5) and pulp involvement (code 6) was notably high, particularly in the lower molars, indicating a considerable burden of untreated decay. (Fig 2)

#### Permanent teeth.

**Fig 3:**
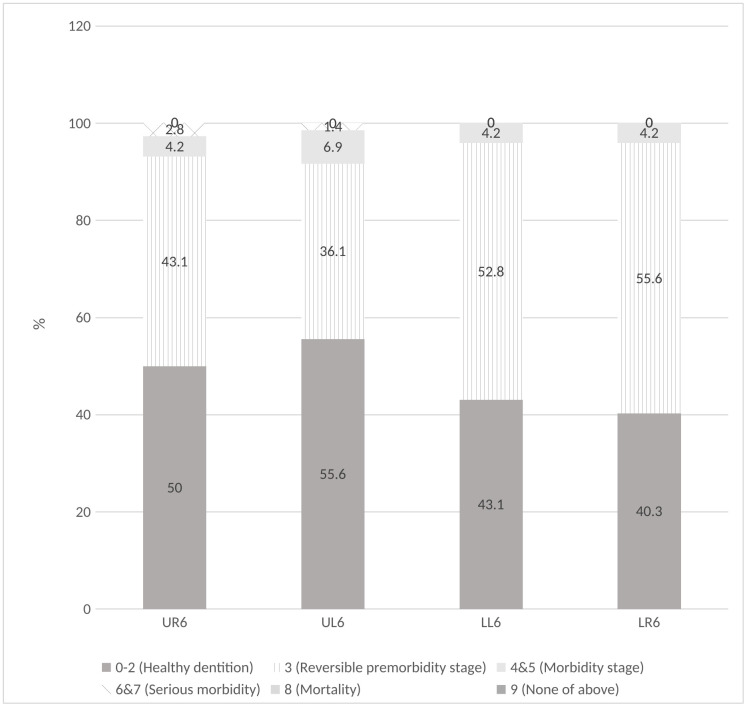
Distribution of the permanent molars’ status based on CAST categories: healthy, pre-morbidity, morbidity, serious morbidity, and mortality.

Among permanent molars, the highest prevalence of healthy teeth (code 0) was observed in the upper left first molar (tooth 26), with 54.2% of cases showing sound surfaces. In contrast, the lower first molars demonstrated the lowest frequency of code 0, each at 38.9%. Enamel caries (code 3) was most commonly identified in the lower right first molar (tooth 46), affecting 55.6% of children. A total of three first permanent molars were classified as having pulp involvement (code 6). Notably, no permanent molars were assigned codes 7 (abscess/fistula) or 8 (extracted due to caries). Additionally, no sealant applications (code 1) were recorded for any permanent molars. (Fig 3)

### Regression analysis

To explore potential predictors of dental caries status as assessed by the CAST index, both unadjusted and adjusted logistic regression analyses were conducted (Tables 5 and 6).

**Table 5:** Association between independent variables and dental caries status of primary teeth based on having a CAST score of ≥3 among orphaned children (n = 72).

Variable	unadjusted			adjusted		
	OR	CI	p-Value	OR	CI	p-Value
Gender	2.82	0.16-47.6	0.47	9	0.03-2323	0.43
Age	0.34	0.057-2.05	0.24	0.19	0.005-7.5	0.37
Educational level	0.51	0.15-1.73	0.28	1.4	0.08-25.8	0.79
History of receiving health education services	1.82	0.1-30.5	0.67	0.33	0.004-28.1	0.63
Tooth brushing	0.69	0.25-1.89	0.47	0.75	0.2-2.81	0.67
Snacks	0.75	0.04-12.6	0.84	5.1	0.061-432	0.47
Knowledge	1.04	0.44-2.45	0.92	0.99	0.11-8.4	0.99
Attitude	1	0.76-1.32	0.97	1.17	0.6-2.3	0.63
practice	0.93	0.57-1.52	0.79	0.67	0.22-2.09	0.49
Logistic regression analysis						

**Table 6:** Association between independent variables and dental caries status of permanent teeth based on having a CAST score of ≥3 among orphaned children (n = 72).

Variable	unadjusted			adjusted		
	OR	CI	p-Value	OR	CI	p-Value
Gender	1.06	0.34-3.3	0.91	2.28	0.3-16.89	0.41
Age	1.4	1.05-1.87	0.02	3.73	0.97-14.35	0.055
Educational level	1.3	0.98-1.83	0.06	0.55	0.14-2	0.38
History of dental visits	0.5	0.16-1.55	0.23	0.3	0.04-2.29	0.24
History of receiving health education services	1.2	0.41-3.48	0.73	7	0.74-65.9	0.08
Tooth brushing	0.72	0.52-0.99	0.04	0.52	0.3-0.88	0.015
Use of fluoride toothpaste	1.46	0.15-14	0.73	2.93	0.16-51.9	0.46
Snacks	1.13	0.39-3.28	0.81	1.25	0.26-6	0.77
Knowledge	0.98	0.71-1.34	0.9	1.45	0.73-2.88	0.27
Attitude	0.92	0.82-1.03	0.14	0.7	0.53-0.93	0.013
practice	0.99	0.84-1.18	0.99	1.23	0.89-1.69	0.19
Logistic regression analysis						

#### primary teeth.

In the unadjusted model, none of the investigated variables demonstrated a statistically significant association with caries status. History of dental visits and the use of fluoride toothpaste yielded extremely high ORs (7020 and 5384, respectively), with p-values of 0.99, likely due to the absence of variance or sparse data in the exposure groups. Similarly, higher oral health knowledge (OR=1.04, 95% CI: 0.44–2.45, p = 0.92) and practice (OR= 0.93, 95% CI: 0.57–1.52, p = 0.79) were slightly associated with lower odds of dental caries, whereas more positive attitudes (OR= 1.00, 95% CI: 0.76–1.32, p = 0.97) showed no meaningful association. However, none of these associations reached statistical significance.

In the adjusted model, the results remained consistent, with no variables showing statistically significant associations with caries status. Gender, age, and educational level remained non-significant predictors. Children who brushed their teeth more frequently showed a tendency toward lower odds of caries (adjusted OR = 0.75, 95% CI: 0.2–2.81, p = 0.67), whereas those who consumed snacks more often tended to have higher odds (adjusted OR = 5.1, 95% CI: 0.061–432, p = 0.47). However, these associations were not statistically significant. Similarly, knowledge, attitude, and practice scores did not demonstrate meaningful associations with dental caries status after adjusting for other variables (Table 5).

#### permanent teeth.

In the adjusted model, gender was positively associated with oral health status, with boys demonstrating higher odds of better outcomes compared to girls, suggesting a potential gender-related difference after controlling for confounders. Age showed a statistically significant association in the unadjusted analysis (unadjusted OR: 1.4; 95% CI: 1.04–1.87, P = 0.02), indicating that older children were more likely to have favorable oral health. This association was borderline significant after adjustment (adjusted OR: 3.73; 95% CI: 0.97–14.35; p = 0.05). Although educational level was marginally associated with oral health in the unadjusted model, this association disappeared in the adjusted model.

No significant associations were observed for history of dental visits or receipt of health education services in either model. However, the latter showed a trend toward significance in the adjusted model, suggesting a potential positive influence of health education. Tooth brushing showed a consistent and statistically significant association with improved oral health outcomes in both unadjusted and adjusted models, underscoring its strong protective effect against dental caries.

Conversely, the use of fluoride toothpaste, snacking frequency, and knowledge scores demonstrated weak, non-significant associations, suggesting that behavioral awareness alone may not translate into healthier practices. Among psychosocial variables, attitude emerged as a significant predictor in the adjusted model (adjusted OR: 0.7; 95% CI: 0.53–0.93; p = 0.013). More positive oral health attitudes were linked to better outcomes, emphasizing the importance of motivational and cognitive factors in shaping oral health behaviors. Practical scores, however, remained non-significant, indicating a potential gap between reported practices and actual preventive behaviors (Table 6).

Table 7 presents the results of unadjusted and adjusted linear regression analyses examining the relationship between the OHI-s index and various demographic, practice, and cognitive variables. In the unadjusted model, several variables showed statistically significant associations with the outcome. Age and educational level were both inversely associated with the outcome, suggesting that older children and those with higher educational levels had better oral health scores. Snacking frequency was also negatively associated with the outcome, indicating that more frequent snacking may be linked to poorer oral health (Unadjusted β = −0.29, p = 0.011). Additionally, oral health practice displayed a significant negative association. This implies that better practice scores corresponded to improved oral health outcomes (Unadjusted β = −0.32, p = 0.006).

**Table 7:** Oral health status based on the OHI-s index and demographic variables, and health practices in orphaned children (n = 72).

Variable	unadjusted			adjusted		
	β	std	p-Value	β	std	p-Value
Gender	0.19	0.2	0.1	0.004	0.24	0.97
Age	−0.27	0.04	0.021	−0.09	0.14	0.8
Educational level	−0.28	0.05	0.014	−0.12	0.15	0.73
History of dental visits	0.15	0.2	0.18	0.06	0.24	0.64
History of receiving health education services	0.06	0.18	0.59	0.27	0.26	0.1
Tooth brushing	−0.21	0.042	0.07	−0.11	0.05	0.42
Use of fluoride toothpaste	−0.03	0.35	0.79	−0.01	0.36	0.93
Snacks	−0.29	0.17	0.011	−0.16	0.2	0.23
Knowledge	−0.19	0.056	0.1	−0.04	0.09	0.81
Attitude	−0.18	0.02	0.12	−0.09	0.03	0.65
practice	−0.32	0.03	0.006	−0.25	0.04	0.11
Linear regression analysis						

After adjustment for potential confounders, most associations lost statistical significance, suggesting that the initial effects were likely influenced by overlapping sociodemographic or behavioral factors. Gender showed a slight positive shift but remained non-significant (adjusted β = 0.004, p = 0.97), indicating minimal contribution to outcome variability. The initially significant associations for age, education, and snacks weakened and became statistically non-significant. Although oral health practice continued to show a negative association with poorer outcomes (adjusted β = −0.25), the relationship did not reach statistical significance (p = 0.11), reflecting a trend toward a protective effect that warrants further exploration. Other variables, such as tooth brushing, knowledge, and attitude, showed no significant associations in either model.

Overall, these findings indicate that factors such as age, educational attainment, snacking habits, and oral health practice may contribute to variations in oral health outcomes; however, their apparent effects may be attenuated or confounded when accounting for other variables in adjusted analyses.

## Discussion

This study examined the knowledge, attitudes, and practices of orphaned children regarding oral health and assessed their correlation with demographic factors and oral healthcare status.

The majority of children (approximately 9 out of ten) had poor DI-s scores, but over four-fifths had good CI-s scores, yielding an overall moderate oral hygiene status. Mean GI values indicated mild gingival inflammation. These results align with Kumari et al. [10] but are more favorable than those of Meshki et al. [25], possibly reflecting differences in welfare and institutional care and access to preventive services.

To our knowledge, and based on a review of the available literature, this study is among the first to apply the CAST index in a population of orphaned children. The findings are consistent with earlier reports indicating poorer oral health among institutionalized children compared with those living in family settings [5,25]. Similar patterns have been described in studies by Booth et al. [26] and Xu et al. [27], who reported higher DMFT/PUFA scores and a higher prevalence of gingival inflammation in socially vulnerable child populations.

The CAST-based assessment enabled a more granular description of lesion severity and distribution. Fewer than one-third of primary molars were classified as sound (code 0), less than one-fifth were restored, and almost half of permanent first molars showed enamel caries (code 3). These findings indicate a substantial level of untreated disease and limited exposure to preventive or restorative dental services in this population. In contrast, no sealants or advanced lesions (codes 7 or 8) were identified. This differs from the results of Babaei et al. [28], who reported a high proportion of healthy permanent molars and frequent use of sealant. The comparable prevalence of dentinal lesions observed in both studies may reflect shared contextual characteristics, including age-related caries progression and socioeconomic deprivation.

No statistically significant associations were found between CAST scores and reported dietary or oral hygiene practices, aligning with the results of Mahboobi et al. [29]. This suggests that caries severity in this population may be more closely associated with socioeconomic conditions than with individual-level behaviors alone. Similar lesion patterns in deprived child populations have been reported by Baginska et al. [22] and Ribeiro et al. [30], supporting the link between deprivation and caries burden. Bhoopathi et al. [31] reported lower DMFT values among older adolescents, which may reflect differences in study populations, settings, or methodological approaches. Evidence from Bhoopathi et al. [31] further suggests that improvements in socioeconomic conditions are associated with better oral health outcomes, while Marandi et al. [32] highlighted the protective associations of maternal education and employment.

Taken together, the present findings indicate that caries patterns among orphaned children are associated with socioeconomic and educational disparities rather than with individual oral hygiene behaviors. Although causal relationships cannot be inferred within a cross-sectional design, the results emphasize the importance of integrating social and structural determinants into oral health promotion and preventive strategies for vulnerable child populations.

The present study revealed significant gaps in oral health knowledge and practices among the participating orphaned children, which suggests individual practices alone cannot fully account for disparities in oral health outcomes. Awareness of the benefits of fluoride-containing mouthwash was particularly low, with only about one-eighth of children demonstrating correct understanding. Similarly, misconceptions about the differences between primary and permanent teeth, as well as the role of bacterial acids in the destruction of dental structures, were prevalent. These gaps highlight the persistence of misconceptions that may compromise preventive oral health practice.

Consistent with Hashemipour et al. [33] and Mohamed et al. [34], participants’ oral health knowledge was moderate to fair. Therefore, targeted education and the urgent implementation of structured oral health education programs are needed to address such misconceptions. Oral hygiene practices were suboptimal: few children reported regular, correct brushing or flossing, and nearly half never rinsed after sweets. This practical pattern mirrors the findings of Das et al. [35] and Kubota et al. [36].

Our finding that oral health knowledge and self-reported practice were inconsistent predictors of dental caries warrants further consideration. Practice items may lack sensitivity, and self-reported measures are prone to social desirability bias, potentially leading to over-reporting. Moreover, even with adequate knowledge or reported positive practice, structural and environmental barriers—such as limited access to dental care, financial constraints, or orphanage care practices—may reduce their impact. These observations align with prior studies [37] emphasizing that effective interventions must address both individual and contextual determinants.

Collectively, these findings underscore the urgent need for structured, context-appropriate oral health promotion in orphanages. Low-cost and culturally adapted interventions—such as visual education, peer-led sessions, and supervised hygiene routines—could help bridge the gap between knowledge and practice. Programs should integrate caregiver involvement and regular dental screening to ensure sustainability and long-term impact. Without such initiatives, the cycle of poor oral health knowledge and inadequate practices in institutionalized children is likely to persist, perpetuating preventable disease burdens into adulthood.

### Strengths

Traditional indices, such as DMFT, PUFA, and ICDAS, have limitations in capturing the full spectrum of caries development, particularly in early stages or for treatment planning [28]. In this study, we used the CAST index, developed by Frencken et al. in 2012, which offers a comprehensive, hierarchical, and clinically relevant framework for assessing dental caries. Its detailed structure makes it especially appropriate for pediatric epidemiological research [38]. The study employed a census-based sampling strategy, ensuring a representative cohort adjusted for socioeconomic background. To reduce inter-examiner variability and enhance data consistency, all clinical assessments were conducted by a single calibrated examiner. Importantly, by applying the CAST index in this context, the study addresses a notable gap in the literature and contributes novel insights into the oral health status of orphan children.

### Limitations

This study has several limitations that should be acknowledged. The relatively small sample size and the fact that participants were recruited from a single city may limit the generalizability of the findings to broader populations. As the study relied on self-reported information to assess oral health practices, responses may have been influenced by social desirability bias, leading participants to overreport positive behaviors. The scarcity of existing research employing the CAST index among orphaned populations posed challenges for comparative analysis. Although oral administration and simplified wording were necessary to facilitate participation among younger children, this approach may have affected the validity and reliability of responses. This limitation should be considered when interpreting the study’s findings. To mitigate these limitations, rigorous calibration and statistical procedures were used to enhance the reliability, and all interpretations were made cautiously within the scope of the sample. Despite these constraints, the study drew upon the most relevant and methodologically comparable literature to contextualize the results. It also provides valuable preliminary insights into the oral health status of this underrepresented group, highlighting directions for future research.

## Conclusions

This study underscores the considerable oral health challenges faced by orphaned children in Karaj, Iran. The high prevalence of enamel caries, lack of restorative and preventive care (including the absence of fissure sealants), and limited knowledge regarding essential dental practices—such as the role of fluoride—collectively reflect unmet oral health needs in this vulnerable population. While oral hygiene indicators, such as the OHI-s and GI, suggest a moderate level of hygiene, the co-occurrence of untreated carious lesions points to deficiencies in early intervention and routine preventive services.

Significant associations between oral health outcomes and variables such as age, grade level, and hygiene practice reinforce the importance of tailored public health strategies. These findings emphasize the urgent need for targeted oral health education and prevention programs within orphanages and similar institutional settings. Policymakers and health professionals should prioritize structured, age-appropriate interventions, including regular dental screenings, oral hygiene instruction, and access to preventive care, to address the oral health disparities identified in this study.
